# Disturbances in White Matter Integrity in the Ultra-High-Risk Psychosis State—A Systematic Review

**DOI:** 10.3390/jcm10112515

**Published:** 2021-06-06

**Authors:** Katarzyna Waszczuk, Katarzyna Rek-Owodziń, Ernest Tyburski, Monika Mak, Błażej Misiak, Jerzy Samochowiec

**Affiliations:** 1 Department of Psychiatry, Pomeranian Medical University, Broniewskiego 26 Street, 71-460 Szczecin, Poland; samoj@pum.edu.pl; 2 Department of Health Psychology, Pomeranian Medical University, Broniewskiego 26 Street, 71-460 Szczecin, Poland; katarzynarek90@gmail.com (K.R.-O.); monika.mak@gmail.com (M.M.); 3 Institute of Psychology, SWPS University of Social Sciences and Humanities, Tadeusza Kutrzeby 10 Street, 61-719 Poznan, Poland; ernest.tyburski@gmail.com; 4 Department of Genetics, Wroclaw Medical University, K. Marcinkowskiego 1 Street, 50-368 Wroclaw, Poland; mblazej@interia.eu

**Keywords:** ultra-high risk, diffusion tensor imaging, DTI, fractional anisotropy, white matter

## Abstract

Schizophrenia is a severe and disabling mental illness whose etiology still remains unclear. The available literature indicates that there exist white matter (WM) abnormalities in people with schizophrenia spectrum disorders. Recent developments in modern neuroimaging methods have enabled the identification of the structure, morphology, and function of the underlying WM fibers in vivo. The purpose of this paper is to review the existing evidence about WM abnormalities in individuals at ultra-high risk of psychosis (UHR) with the use of diffusion tensor imaging (DTI) available from the National Center for Biotechnology Information PubMed (Medline) and Health Source: Nursing/Academic Edition databases. Of 358 relevant articles identified, 25 papers published in the years 2008–2020 were ultimately included in the review. Most of them supported the presence of subtle aberrations in WM in UHR individuals, especially in the superior longitudinal fasciculus (SLF), the inferior longitudinal fasciculus (ILF), and the inferior fronto-occipital fasciculus (IFOF). These alterations may therefore be considered a promising neurobiological marker for the risk of psychosis. However, due to methodological discrepancies and the relative scarcity of evidence, further investigation is called for, especially into connectome analysis in UHR patients.

## 1. Introduction

Psychosis is a state in which a patient experiences gross impairment of reality testing, manifesting in the form of disturbances in perception (e.g., hallucinations or hallucinoids) and thinking (e.g., delusions or obsessions) as well as disorganization symptoms, and where the patient lacks the capacity to gain insight into the pathological nature of these occurrences. Psychotic experiences may occur in the course of various mental disorders, including schizophrenia spectrum disorders, mood disorders, certain personality disorders, substance use disorders, as well as in neurological conditions (e.g., Alzheimer’s dementia or Huntington’s disease dementia). Schizophrenia (a chronic mental illness that leads to severe impairment in cognitive and social functioning) was first observed in the 19th century by Emil Kraepelin, who referred to it as “dementia praecox” [1]. Cognitive dysfunction includes deficits in attention, executive functions, memory, facial recognition, as well as disturbances in social cognition and day-to-day functioning [2,3,4,5]. Since it is known to most severely affect people of working age, leading to loss of employment, stigmatization, and isolation, schizophrenia also imposes a serious financial burden on both states and those affected by it [6,7]. There are additional illness-related costs incurred by its common comorbidities: analyses show that schizophrenia patients are at a 2–3 times greater risk of mortality [8,9] and a significantly greater risk of diabetes (15% of patients with schizophrenia compared to 6% in the general population) and metabolic syndrome [10]. Therefore, it is crucial to develop effective measures for early recognition and intervention. The most critical prognostic factor is the duration of untreated psychosis (DUP; i.e., the time that has elapsed from symptom onset to the initiation of treatment): evidence suggests that greater positive symptom severity and poorer global functioning are associated with longer DUP [11].

For over 20 years, efforts have been made to investigate the ultra-high-risk state for psychosis (UHR), also known as the “prodromal”, “at-risk”, or “clinically high-risk” state [12]. To avoid confusion, in this paper, we will only use the abbreviation UHR. Identifying people at risk of developing full-blown psychosis and providing them with the necessary health care could significantly reduce the DUP and, thus, radically improve their prognoses.

To date, various tools to screen for psychosis have been developed, including the Community Assessment of Psychic Experiences (CAPE-42), the PRIME Early Psychosis screening test, the Prodromal Questionnaire (PQ), Prodromal Questionnaire—Brief version (PQ-B), the Adolescent Psychotic-like Symptom Screener (APSS), and the Psychosis-like Symptoms Interview (PLISKi), to name but a few. They are, however, burdened with numerous limitations [13]. The questionnaires differ in terms of their sensitivity (67–100%) and specificity (39–100%), administration (self-assessment vs. interview with a healthcare specialist), number of assessed parameters (6–92), and, finally, their purpose: some are designed to serve as general population screeners, others to identify UHR among help-seeking individuals. In addition, structured questionnaires have been developed to either screen for UHR syndromes, such as the Structured Interview for Prodromal Symptoms (SIPS) [14,15] or the Comprehensive Assessment of At-Risk Mental States (CAARMS) [16], or for the early signs of psychosis, the Bonn Scale for the Assessment of Basic Symptoms (BSABS) [17] and the Basel Screening Instrument for Psychosis (BSIP) [18].

Nevertheless, none of the existing screening tests provide prognostic certainty of developing psychosis, and so it is all the more important to search for biological markers (i.e., indicators of its physiological and pathological underpinnings or response to treatment; [19]) that can be objectively measured and assessed, thus improving diagnostic certainty.

To date, candidate biomarkers for psychosis have been sought in several domains, including genetics, biochemistry, neurophysiology, neuropsychology, and neuroimaging [20]. Given the dynamic development of modern neuroimaging methods and consistent reports on the mediating role of white matter (WM) in the etiopathology of schizophrenia [21], the magnetic resonance tractography technique (diffusion tensor imaging; DTI) seems to show particular promise. This non-invasive technique allows in vivo imaging and tracking of nerve fibers and visualization of Brownian motion (microscopic chaotic movements of water molecules in extracellular space; [22]). In a magnetic field, these molecules arrange themselves along or across the surrounding biological structures. Taking advantage of the diffusion-sensitive magnetic resonance imaging sequence, it is possible to evaluate brain tissue characteristics via the distribution of three-dimensional diffusion directions in different portions of the brain.

The DTI indices used to assess the organization and integrity of white matter nerve fibers and identify pathways of nerve fiber tracts include fractional anisotropy (FA), which ranges from 0 (perfectly isotropic diffusion) to 1 (extremely anisotropic diffusion); mean diffusivity (MD), which represents the general magnitude of diffusion at a selected point in space; axial diffusivity (AD), which reflects diffusivity parallel to fiber tracts; and radial diffusivity (RD), which describes the magnitude of water diffusion perpendicular to the tracts. According to Andreasen’s neurodevelopmental model of schizophrenia [23], disturbances in the integrity of nerve fibers (i.e., the etiology of the psychopathological symptoms of schizophrenia) may emerge in response to abnormalities at various stages of life, from prenatal development to adulthood.

Previous research has focused mainly on patients diagnosed with schizophrenia, describing, inter alia, alterations within the uncinate, superior, and inferior longitudinal fasciculus (ILF), arcuate fasciculus, cingulate gyrus (CG), and the corpus callosum (CC; [24]). Over the past 20 years, there has been growing interest in the analysis of white matter in UHR populations. Nevertheless, to the best of our knowledge, no meta-analysis on the subject has been published to date. There are three systematic reviews that include papers published before 2015 [25,26,27] and several selective reviews. However, none of these seem to offer an extensive analysis of DTI indices in UHR individuals (other than FA). Moreover, the links between WM integrity and psychopathological symptoms seem to have been severely underinvestigated. This is all the more surprising as comparisons of outcomes in UHR individuals help us gain a better understanding of the nature of psychosis and organize a support system for those at risk of developing it, which offers particular benefits not only in the form of potentially reduced risk of conversion to full-blown psychosis and lower incidence of other mental disorders (e.g., anxiety or depressive disorders that are often comorbid to UHR states; [28]) but also attributable to earlier and more effective provision of care to individuals with persistent or recurrent intermittent psychotic symptoms, thus improving the quality of their social functioning. Even a slight improvement of prognostic certainty or prognosis could enable earlier implementation of effective treatment and improved quality of life. Unfortunately, we still lack sufficient data on specific white matter changes in UHR individuals; therefore, before assessing whether they could be a potential biomarker, a comprehensive literature review should be conducted. Given the aforementioned limitations and the paucity of comprehensive analyses in the available review papers, we formulated the following objectives: (1) to perform a systematic review of research results on the assessment of abnormalities in WM integrity in UHR individuals using DTI and (2) to evaluate the available data on the relationship between WM integrity and psychopathological symptoms in UHR individuals.

## 2. Materials and Methods

### 2.1. Literature Search

A systematic review of studies using DTI imaging in UHR patients was conducted after pre-registration in PROSPERO (CRD42020198162). Details of the protocol can be accessed at https://www.crd.york.ac.uk/PROSPERO/display_record.php?ID=CRD42020198162 (accessed on 3 June 2021). The search strategy was prepared using the PICO framework.

The search was performed on the PubMed (Medline) databases of the National Center for Biotechnology Information and Health Source: Nursing/Academic Edition; they included the following search terms in various combinations: “DTI”, “diffusion tensor imaging”, “white matter”, “UHR”, “ultra-high risk”, “CHR”, “clinical high risk”, “psychosis”, “ARMS”, “at-risk mental state”, and “schizophrenia prodrome”. UHR was understood as referring to patients who met the diagnostic criteria for the high-risk state for psychosis, namely: (1) attenuated psychotic symptoms; (2) brief limited intermittent psychotic symptoms; or (3) functional deterioration (30% drop in score on the Global Assessment of Functioning within a year) and genetic risk (family history of psychotic disorders or personal history of schizotypal personality disorder).

### 2.2. Inclusion and Exclusion Criteria

We included original research papers published before May 2020 that met the following criteria: (1) the study sample was composed of UHR individuals; (2) the diagnosis of UHR was based on one of the structured diagnostic questionnaires (SIPS, CAARMS, BSABS, and BSIP); (3) the neuroimaging technique used was DTI, and at least one of the most commonly used DTI parameters were described: FA, AD, MD, or RD; (4) healthy controls (HC) were included; and (5) the publication was in the English language.

Exclusion criteria were: (1) non-original data (systematic or non-systematic reviews, conference abstracts, case studies, etc.); (2) non-UHR individuals (or studies using a mixed sample of UHR patients and patients with a different diagnosis); (3) exclusive use of a genetic high-risk population; (4) research using questionnaires other than those specified; (5) different neuroimaging techniques (no white matter assessment); (6) the required DTI parameters not having been analyzed; and (7) language other than English.

### 2.3. Data Extraction

**Figure 1:**
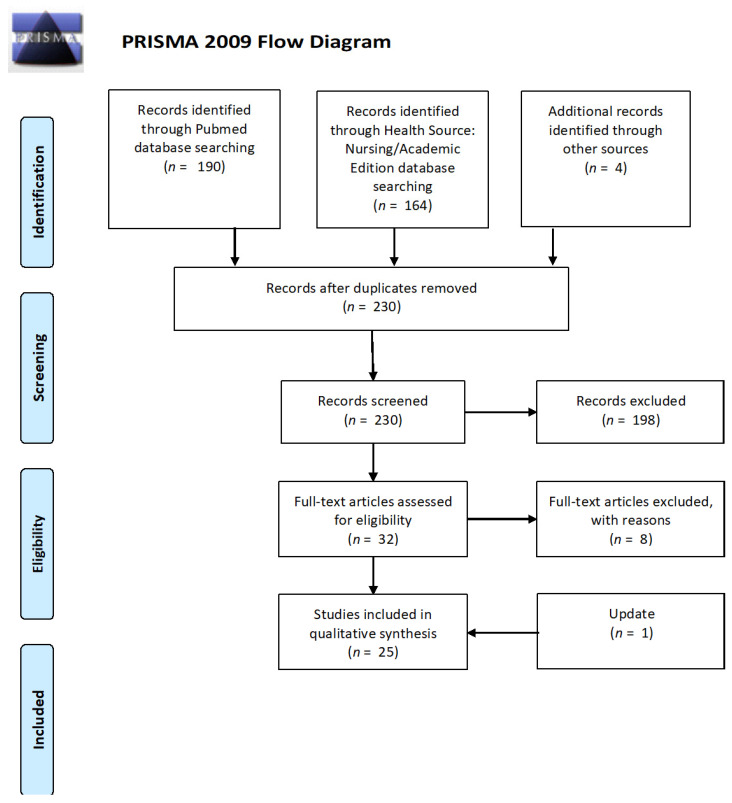
PRISMA flow diagram [29].

Two researchers (KW and ET) independently screened records to be included. Any discrepancies were resolved by consensus. Search results were screened for eligibility in 2 stages: first by title and abstract, then by full-text. Reasons for exclusion were recorded for each excluded study. In addition, a manual search of the references from the articles obtained from the databases was performed. After excluding duplicates, 230 studies were identified, and a decision was made to include a further 4 publications found among the references of the initially identified papers. Screening the titles and abstracts, we further excluded literature reviews (20), studies not involving UHR populations (154), studies on samples including individuals with an increased genetic risk of developing psychosis only (13), research protocols (1), studies using other classification tools (4), case studies (3), one letter to the editor (1), and conference abstracts (2). The remaining 32 papers were read in full; of these, we ultimately excluded: one study in which the UHR individuals were a subsample of a different study group (1), ones in which DTI was not the primary imaging technique or that analyzed non-relevant DTI parameters (4), a letter to the editor (1), one study in which only gray matter was tested (1), and one in which major discrepancies concerning the study group appeared (1). Thus, there were 24 studies in the final analysis. The following data were extracted from the included studies: authors, year of publication, methodology used in the original analyses (study design; tools used to measure symptom severity and level of functioning; and sample size and characteristics, i.e., age range, sex distribution, substance use, and medication status), the data needed for the assessment of research quality, and any coefficients/descriptive statistics required to analyze the relationship between white matter fibers and symptom severity and level of functioning. The review was updated in September 2020, and one more article meeting the aforementioned criteria was included in the qualitative synthesis. The article selection strategy is presented in Figure 1.

### 2.4. Quality and Risk of Bias Assessment

The quality and risk of bias assessment was conducted by the two researchers independently (KW and ET). The criteria of Kmet, Lee, and Cook (2004) were used for the quality evaluation of each study. The following quality criteria were used: (1) sufficient description of the research questions, (2) clarity and appropriateness of used design, (3) sufficient description of the methodology of participant selection or source of information, (4) sufficient description of sample characteristics, (5) definitions of measurements, (6) appropriate sample size, (7) justification of analytic methods used, (8) control for confounding factors, (9) sufficient detail of reported results, and (10) support for conclusions. A three-point rating scale was used for qualitative assessment (2—yes; 1—partial; 0—no). Unsuitable items were labeled “n/a” and excluded from the summary score (Kmet et al., 2004). We assumed a cut-off point for the inclusion of 65% (indicating a moderate-to-high quality) of the potential maximum score, which is recommended in the literature.

## 3. Results

### 3.1. Search Results

Table 1 gives a summary of the 25 identified articles published between 2008 and September 2020. In 18 studies, the UHR diagnosis was based on the SIPS or the Scale of Prodromal Symptom (SOPS), in 5 it was based on the CAARMS, 3 studies defined UHR as the presence of at least two “basic symptoms” according to the BSABS, while in the 2 remaining studies the BSIP and the Wisconsin schizotypy scales were used to determine UHR in the sample [30,31]. Five studies relied on more than one structured diagnostic interview.

The diagnostic methods used combined the following approaches: region of interest (ROI) analysis, which enables the manual tracking of the degree of diffusion in specific locations of individual nerve pathways; voxel-based analysis, which is the automated, simultaneous assessment of the degree of diffusion in every voxel of a whole-brain data set; and tract-based spatial statistics (TBSS), a technique that projects individual DTI parameters onto an averaged white matter skeleton representing nerve fiber centers.

Most of the selected papers analyzed association fibers (superior longitudinal fasciculus; SLF; inferior longitudinal fasciculus; inferior fronto-occipital fasciculus; IFOF; uncinate fasciculus; UF; cingulum bundle; CB; external capsule; EC; and the internal capsule; IC), commissural fibers (corpus callosum), and projection fibers (anterior thalamic radiation; ATR; posterior thalamic radiation; PTR; and corticospinal and cerebellospinal fibers).

#### 3.1.1. Study Characteristics

The sample sizes of the 25 studies varied from 10 to 116 UHR individuals, with a total number of 881 participants. The mean ages of patients ranged from 12.2 to 25.9 years, and all except two articles included both male and female participants.

#### 3.1.2. Quality Assessment and Risk of Bias

The quality of the included articles was assessed by summarizing the risk of bias (see Supplementary Material). After the two researchers independently performed the assessment, the scores were summed, and the mean value was calculated. All of the studies achieved the cut-off point of 65% and were included in the review.

### 3.2. Comparison of DTI Parameters

#### 3.2.1. Fractional Anisotropy

The only DTI parameter described across all reviewed papers was FA, although the analyses yielded conflicting results. Most studies reported an initial decrease in FA in cellular tissue [32] or FA in the frontal or temporal lobes [32,33,34,35,36,37,38,39,40,41,42,43,44,45,46]; specifically, bilaterally in the upper frontal lobes [33], corticospinal pathway [35], forceps minor [46], in the SLF [33,34,38,41,42,46], ILF [33,34,35,37,40], IFOF [33,34,35,37,40,46], CB [36,37,42,46] UF [46], CC [34,39,44,46], PTR [34], ATR [40,41,42,46], and EC and IC [34,45]. In six studies, no differences were found between UHR and healthy controls at baseline [47,48,49,50,51,52], while in three studies, baseline FA was higher in the UHR group in numerous regions of the brain, mostly the right ATR, left IFOF, SLF, UF and forceps minor [53], as well as in the hippocampal-thalamic [54] and cerebellar-thalamic tracts [54]. In two of the studies, reduced FA was described only in UHR individuals who developed psychosis (UHR-P), in the corticospinal tract, forceps major, SLF, ILF, IFOF, CC, ATR [43], and PTR [55].

In 16 studies, UHR patients were observed in terms of conversion to psychosis, but only 3 reported significant differences from HC in terms of initial FA, which was reduced in the left SLF, ILF, IFOF, forceps major, CC, left corticospinal tract, left ATR [43], PTR [55], and CC [44]. Paradoxically, reduced FA values in the CC were found in non-converters (UHR-NP), both at baseline (the genu and body of the CC) and at a 52-month follow-up (the body of the CC) [44]. Another two studies described differences in baseline FA in UHR-P compared to UHR-NP: reduced in the forceps minor [46], in the right UF, IFOF, and SLF (laterally to the putamen), in the left superior temporal lobe (SLF, ILF, IFOF); and higher in the left middle temporal lobe (PTR, IFOF, ILF) [33], or only in the PTR [55]. Three studies found no baseline differences in FA values between UHR-P and UHR-NP [32,34,51], but one of them described a drop in FA at a 28-month follow-up in the anterior limb of the left internal capsule, left superior corona radiata, and body of the CC [34]. In the remaining papers, the number of conversions of UHR into full-blown psychosis was too small to draw statistically significant conclusions.

#### 3.2.2. Radial Diffusivity

RD was another DTI parameter examined (nine studies); it had higher baseline values in UHR participants relative to HC participants across the whole brain [47], in the right inferior cerebellar peduncle, bilaterally in the medial lemniscus, right corticospinal tract, middle cerebellar peduncle, superior cerebellar peduncle, cerebral peduncle, left UF, left ILF, bilaterally in the IFOF and EC, right IC, in the retrolenticular part of the left IC, the splenium and body of the CC, bilaterally in the SLF, posterior and superior corona radiata [34], PTR [34,48], and CG [36]. In one study, higher RD was observed only among UHR-P in the body and splenium of the CC, forceps major, ATR, cingulum, and the right corticospinal tract [43]. There were also reports of no significant differences in RD [40,46,52] and RD in cellular tissue [32] in UHR individuals, although in one of these studies, after a multivariate partial least squares correlation (PLSC) analysis, RD values turned out to be significantly higher in many brain regions [40].

#### 3.2.3. Axial Diffusivity

AD analysis (8 studies) also yielded some conflicting results, suggesting: no significant differences between UHR and HC [36,40,48] or between UHR-P and HC [43]; a baseline drop in AD values in the middle cerebellar peduncle, left corticospinal tract [34], CG and CC bilaterally [46]; but also elevated AD globally [47] or in the right UF, right EC, retrolenticular part of the right IC, right fornix, right ILF and IFOF, and CC [34]. In one study, after PLSC analysis, a significant reduction in AD was observed in numerous regions of the brain [40], while another one found a reduction in AD in cellular tissue, which, however, became statistically insignificant after TBSS [32].

#### 3.2.4. Mean Diffusivity

MD values were compared in five studies, yielding inconsistent results: no significant differences between UHR-P and HC [43] or between UHR and HC [46]; higher MD in UHR relative to HC in the right hemisphere (mainly the SLF, superior and posterior corona radiata, PTR, posterior IC, body and splenium of the CC, CG, anterior limb of the IC, and cerebral peduncles [48]) and globally [47]; and lower in many regions of the brain, especially the left SLF and ILF, IFOF, and right ILF [53].

#### 3.2.5. Combination of DTI Indices

A total of eight studies reported FA, AD, and RD, with six of them indicating decreased FA in various WM areas and two indicating nonsignificant FA differences. Decreased FA value was not consistently connected with changes in AD or RD.

#### 3.2.6. Other Parameters

Only one study described the mode of anisotropy (MO), finding no significant differences between UHR and HC [40], with another study identifying elevated traces of the diffusion tensor in CG [36].

### 3.3. DTI Indices and Psychopathological Symptoms

Most analyses concerned the relationship between FA and symptom severity, measured with the use of the available structured interviews: SIPS/SOPS, CAARMS, Positive and Negative Syndrome Scale (PANSS [56]) and the Scale for the Assessment of Negative Symptoms (SANS [57]). Negative correlations were found between symptom severity and FA in the left IFOF, left UF, and left ATR [46]. Negative correlations were also found between positive symptom severity and FA in the cortico-striatal pathways of the limbic network [45], right superior temporal lobe in UHR, and left middle temporal lobe in UHR-P [33]. A link was found between reduced positive symptoms and increased FA in the CC in UHR-NP [39]. There are also reports of correlations between positive symptom severity and FA in the right and left hemispheres [54], right SLF [53], and FA alterations in the thalamo-motor tract [58]. Only two studies reported a correlation between FA and negative symptom severity in the right ILF and hippocampus [38] and the genu of the CC [44]; another study found a relationship between deficit symptoms severity and FA alterations in the left IFOF, ATR, and SLF [41]. No significant correlations between baseline FA values and positive and deficit symptoms were described in three papers [32,41,47], the last of which also found no association of symptoms with FA, AD, and RD in cellular tissue or free water (FW).

### 3.4. DTI and Socio-Cognitive Functioning

Positive correlations were found between occupational and social functioning, measured with the Global Assessment of Functioning Scale (GAF [59]) and the Social and Occupational Functioning Assessment Scale (SOFAS [60]), and FA in the right ATR [38] and left ILF [38,40]; AD in the left IFOF and right SLF; and MO in the right IFOF, left SLF, and right ATR [40]. One study also showed a positive correlation between FA in the left ILF and left IFOF and executive functions [35] measured with the Delis-Kaplan Executive Function System battery (D-KEFS [61]). Negative correlations between social functioning and FA as well as FA, AD, and RD in cellular tissue were described in one study, although after TBSS, only the relationship with FA in cellular tissue remained statistically significant [32].

Only one observation concerned the presence of the neurological soft signs measured with the Neurological Evaluation Scale [62] in UHR, suggesting that they are significantly more common in this population [49].

## 4. Discussion

Our systematic review of the extant literature focused on investigating the existence and location of disturbances in WM tracts in UHR individuals with the use of DTI. After screening titles, abstracts, and full texts, removing duplicates, and risk and bias assessment, 25 studies were included in the analysis. Most of them supported the presence of subtle aberrations in WM tracts connecting the frontal and temporal lobes. Two specific objectives were formulated in the preparation process. The present review also highlighted key areas for further research.

The first aim of this paper was to systematically review research results on the assessment of WM integrity alterations using DTI in UHR individuals. Most of the cited studies indicate the existence of subtle abnormalities in white matter, although data concerning their location and relationship with psychopathology remains inconsistent. The most frequently mentioned are the white matter tracts connecting the frontal and temporal lobes and their connections, especially the SLF [33,34,38,41,42,43,46,47,48,53], ILF [33,34,35,40,43,53], and IFOF [33,34,35,37,40,43,46,47,53], which is consistent with other studies on schizophrenia [63]. Comparison of DTI indices between schizophrenia patients and UHR individuals is depicted in Table 2. Only 4 out of 25 studies showed no significant differences between UHR individuals and HC in the tested parameters. The most frequently analyzed DTI measure was FA, in which 16 studies described a decrease, 3 studies described an increase, and 6 described no significant differences relative to HC.

WM integrity disturbances are also reported in healthy populations in the process of normal aging, which translates into the deterioration of cognitive and executive performance [64,65]. Descriptions of similar symptoms in UHR, and therefore poorer test scores in intelligence, verbal and visual memory, attention, and working memory [66], prompted the search for their underlying causes in the brain’s WM connectivity. WM disturbances are described in most of the studies cited in this review, and the use of DTI allows us to distinguish UHR from HC. This is further supported by findings from research using machine learning and support vector machines, whose algorithms, based on DTI parameters, enabled correct identification of both groups with almost 66% accuracy (*p* < 0.05); although, interestingly, DTI alone failed to differentiate between UHR and first-episode psychosis (FEP) individuals [67]. Furthermore, WM abnormalities have been described in the genetic risk groups [68,69,70,71], although there are conflicting reports about this [72].

Our second aim was to evaluate the available data on the relationship between WM integrity and psychopathological symptoms in UHR individuals. The only parameter reported to significantly correlate with psychopathology was FA. In most studies, this relationship concerned positive symptoms, although both positive and negative correlations with FA were described. However, findings on alterations within specific WM bundles are inconsistent and should be interpreted with caution. The scarcity of evidence on the effect of WM abnormalities on socio-cognitive functioning prevents the drawing of any definite conclusions in this area.

Differences in DTI indices may be due to the symptom diversity presented by UHR individuals and the relationship of specific bundles or brain structures with particular symptoms, as is observed in schizophrenia [73,74,75]. In addition, symptom severity and the high heterogeneity of respondents may also play a role. Indeed, the definitions of UHR populations comprise individuals with poorly expressed or fully developed, but transient, psychotic symptoms as well as those at genetic risk or with schizotypal disorder combined with deterioration in social functioning, which may be partly responsible for the different transition rate risks (e.g., 39% in the BLIPS subgroup versus 19% in the APS group at 24 months) [76].

The relative scarcity of UHR-P individuals in studies is also quite important. Due to the small sample size in most analyses, the results cannot be extrapolated to populations who are really at risk of developing full-blown psychosis. The initially available evidence suggested that 22–36% of UHR individuals convert to psychosis after 1–3 years [77], but the transition rates have been declining in more recent studies and vary between 16.9% after 2 years [78], 24% after 3 years [79], and 14.6% over a median follow-up time of 4.8 years [80], depending on the research; hence the need to conduct research on a large number of at-risk patients, possibly separately for the BLIPS, APS, and GDR subgroups. Analyses comparing UHR not only with HC but also FEP individuals or those recently diagnosed with schizophrenia suggest similar or less severe WM alterations in UHR relative to the other groups [34,43,48,53,81,82], which suggests that they may occur even prior to the onset of illness. The very process of identifying UHR based on the use of structured interviews (SIPS, CAARMS) assumes that symptoms can be qualitatively assessed and that there exists a cut-off point above which symptoms can be considered psychotic and, if the duration criteria are met, the patient is thus diagnosed with acute psychosis, supporting the concept of a “psychosis continuum” [83,84].

Although quality assessment and risk of bias were performed using the criteria of Kmet, Lee, and Cook (2004) and all of the selected studies achieved the cut-off point of 65% and were included in the review, we did not analyze all possible variables, such as, for example, dropout during follow-up assessment.

However, before any firm conclusions can be drawn, a substantial number of limitations should be considered. First of all, the biggest issue is the relatively small number of studies on UHR populations. This is due, inter alia, to the low reporting rate of people who develop initially unspecified (psychotic) symptoms that spontaneously subside or only occur intermittently. It is also important to not discount the fact that many people may not be aware of where and how to seek help or fear the stigma related to psychiatric treatment. Likewise, the recruitment method used in a given study (in schools, hospitals, through advertisements, etc.) may affect the degree of UHR identification or reporting, which will largely depend on symptom severity or level of personal distress and may be responsible for different transition rates. Studies show a lower pretest risk of psychosis in research targeting the general population when compared to those focused on mental health institutions [85]. Additionally, it is undeniable that there is increased awareness of UHR symptoms and faster referral to mental health professionals trained in recognizing and assessing the risk of psychosis [86]. All these factors contribute to the study groups having low representativeness, preventing inferences from being made and, consequently, making it difficult to compare studies and draw definite conclusions.

Secondly, the age of the respondents is worth remarking upon. Although most articles examined young adults, there were also studies on children or adolescents [35,37] who are at risk of developing symptoms and corresponding WM alterations in the future. Comparisons of individuals of different ages and at different stages of symptom development may therefore lead to premature or incomplete conclusions due to different levels of both white and gray matter maturation (i.e., myelination or synaptic pruning) [87,88]. Brain development is a continuous and dynamic process; therefore, WM alterations might be a prolongation of a developmental process, as the neurodevelopmental models suggest, or a completely new trait appearing at a specific point in time [89]. This highlights the need to establish a uniform timeframe and long-term periodic evaluation principles for the observation of people at risk of psychosis and unfortunately makes drawing any final conclusions at this stage of the research impossible.

Thirdly, sex differences also seem to be important. For many years, the literature has analyzed the role of sex in alterations in the brain structure of schizophrenia patients, age of onset of the disease, its course [90], as well as differences in WM structure. Although the results are still inconclusive [91], we cannot rule out that the higher prevalence of men in most of the analyzed studies may have had a significant effect on the findings, which could affect the objectivity of this analysis.

Fourthly, at-risk populations are exposed to much lower lifetime doses of antipsychotic drugs, which potentially impact WM structure [92]. However, in most of the reviewed studies, antipsychotic treatment was not an exclusion criterion, so conclusions in this regard seem to be unfounded.

Fifthly, there are the limitations of DTI and different methods of data collection. For starters, not having DTI acquisition parameters identical across all the participants raises the question of whether the results can be compared at all. Moreover, concentrating on FA, which might be enhanced due to increased AD, restricted RD, or both, may lead to some discrepancies between results, and therefore, analysis of both FA, AD, RD should be considered in all studies. Methodological differences also meant that some studies relied on manual searches for evidence in support of initial hypotheses within individual nerve pathways, while others used automated methods to scan the whole brain. Such discrepancies, as well as the fact that some observations focused only on specific nerve bundles, neglecting others, may lead to strikingly inconsistent results. In addition, the extent to which the methodological factors of individual analyses (such as resolution being too low or the effect of artifacts on DTI imaging. Due to sensitivity to movement, it is crucial to control the scans for head motion, which was not performed in all studies) affects the quality of collected data remains largely unknown.

Finally, a systematic review itself is not an error-free method. The search for publications was limited to one database, so it is possible that not all available studies on the subject were included in this review. Moreover, due to the significant diversity of the analyzed parameters, research methodology, localization of changes, symptomatology, and described correlations, not all available data were considered in the review.

## 5. Conclusions

This systematic review indicates that DTI is a useful tool for assessing WM abnormalities, although its clinical utility in the assessment of discrete alterations is still limited. Studies that use DTI in UHR populations suggest the presence of subtle WM abnormalities prior to the onset of full-blown psychosis, but reports on their severity and location differ.

The most common reports concern aberrations resembling those typical of schizophrenia, specifically: reduced FA in the frontal and temporal lobes as well as in the connections between them (especially the SLF, ILF, and IFOF). Based on the analyzed studies, no clear conclusions can be drawn regarding the relationship between DTI and psychopathological symptoms or socio-cognitive functioning; although some authors suggest correlations between FA and positive symptom severity, such reports should be treated with caution. Differences in the reviewed studies may be due to various methodological factors, including differences in age, sex, clinical presentation, or the drugs and psychoactive substances used by respondents. To draw definitive conclusions as to whether DTI parameters allow for the identification of UHR requires long-term prospective observations on a large group of respondents with the use of a uniform methodology. Future efforts should focus on the search for diverse biological markers of UHR, such as the structure of nerve bundles, functional connectivity, and genetic and stem-cell-related factors. In addition, as most DTI studies focus on particular connections or brain areas theoretically related to schizophrenia symptoms, it would significantly improve the connectome analysis of the brains of individuals at UHR if comparisons were made not only with healthy controls but also with individuals already diagnosed with schizophrenia or chronic schizophrenia. This would not only increase the chance of locating the WM abnormalities but also improve the test-retest reproducibility of the method used.

## Figures and Tables

**Table 1:** Search results of changes in diffusion parameters in the ultra-high-risk psychosis state.

No.	Authors	Sample	Mean Age	Sex (M/F)	UHR Diagnostic Tool	DTI Parameters (Field Strength, TR—Repetition Time, TE—Echo Time, Number of Slices, Matrix Size, Voxel Size)	Motion Control Reported (Y—Yes, N—No)	Type of Study	WM Alterations UHR vs. HC	WM Association with Psychopathology
1	Peters et al., 2008	10 UHR 10 HC	21.2 ± 3.0 21.1 ± 2.8	10/0 10/0	SIPS, 2 BS	3 T; TE = 94 ms, TR = 4831—6248 ms image matrix 256 × 256, resolution 3 × 3.5 × 2.2 mm^3^	N	Cross-sectional	No significant differences in FA	
2	Karlsgodt et al., 2009	36 UHR 25 HC	17.02 ± 3.37 17.96 ± 3.40	27/9 12/13	SIPS	1.5 T; TR = 9.5 s, TE = 77 ms, 75 contiguous 2 mm slices, matrix 128 × 96, voxel size 2 × 2 × 2 mm^3^	Y	Longitudinal	↓FA in SLF (fronto-parietal junction) no age-related ↑ FA in hippocampus (temporal lobe) or ILF	Correlation between FA in right ILF and right hippocampus and negative symptoms, but no positive symptoms ↓ FA in the hippocampus and ILF was a predictor of deterioration in social functioning
3	Peters et al., 2010	17 UHR 7 UHR-P 10 UHR-NP 10 HC	22.6 ± 3.9 21.2 ± 3.2 21.1 ± 2.8	7/0 10/0 10/0	SIPS, 2 BS	3 T; TR = 8872 ms, TE = 51 ms, 48 continuous slices, slice thickness 3 mm, acquisition matrix 112 × 112; voxel size 2 × 2 × 3 mm^3^	N	Longitudinal	No significant differences in FA	
4	Bloemen et al., 2010	37 UHR 10 UHR-P 27 UHR-NP 10 HC	20.7 ± 4.3 18.9 ± 4.0 22.7 ± 3.9	8/2 18/9 8/2	SIPS	3 T, 48 continuous 3 mm slices, 2 × 2 × 3 mm^3^	N	Longitudinal	↓ FA bilaterally in the superior frontal lobes (SLF, ILF, IFOF) UHR-P vs. UHR-NP: ↓ FA laterally to the right putamen (in UF, IFOF, SLF) ↓ FA in the left superior temporal lobe (SLF, IFO, ILF) ↑ FA in the left medial temporal lobe (PTR, IFO, ILF)	Lower FA in the right upper temporal lobe correlated with greater positive symptom severity Lower FA in the left medial temporal lobe was associated with greater positive symptom severity in UHR-P
5	Jacobson et al., 2010	11 UHR 14 HC	12.2 ± 0.6 12.5 ± 0.4	4/7 3/11	APSS, SIPS	3 T; TR = 12,343 ms, TE = 52 ms, matrix size 150 mm, voxel size 0.9 × 0.9 × 0.9 mm^3^	Y	Cross-sectional	↓ FA in IFOF ↓ FA in the CG (left parahippocampal gyrus) ↓ FA in ILF (left superior temporal gyrus)	
6	Carletti et al., 2012	32 UHR 8 UHR-P 24 UHR-NP 32 HC	23.4 ± 3.8 25.9 ± 5	19/13 28/5	CAARMS	1.5 T; RT = 15 R-R intervals; TE = 107 ms; 60 contiguous 2.5 mm slices, matrix size 96 × 96, voxel size 1.875 × 1.875 × 2.5 mm^3^	Y	Longitudinal	↓ FA in left SLF, ILF, IFOF, PTR, retrolenticular IC, splenium and body of CC, in the left posterior and superior corona radiata ↓ FA in the right EC, retrolenticular part of the right IC, right posterior corona radiata ↑ RD in the right inferior cerebellar peduncle, bilaterally the medial lemniscus, in the right corticospinal tract, in the middle cerebellar peduncle, superior cerebellar peduncle, cerebral peduncle, left UF, left ILF, left IFOF, bilateral EC, right IC, retrolenticular left IC, right IFOF, bilateral PTR, splenium and body of CC, bilateral SLF, posterior and superior corona radiata ↓ AD in the middle cerebellar peduncle, left corticospinal tract ↑ AD in the right UF, right EC, retrolenticular part of the right IC, right fornix, right ILF and IFOF, splenium and body of CC No significant baseline differences between UHR-P and UHR-NP After 28 months: ↓ FA in UHR-P versus UHR-NP in the left anterior limb of the IC, left part of corona radiata, frontal part of CC	
7	Mittal et al., 2014	33 UHR 35 HC	18.52 ± 2.06 17.77 ± 2.71	20/13 15/20	SIPS	3 T; TR = 9600 ms; TE = 86 mm; 72 slices; voxel size 2 × 2 × 2 mm^3^	Y	Longitudinal	No significant baseline differences in the cerebellothalamic tracts ↓ FA in the cerebellar-thalamic tracts after 12 months (in HC: ↑ FA)	UHR: significantly more neurological soft signs
8	Epstein et al., 2014	21 UHR 55 HC	16.1 ± 3.3 16.5 ± 2.6	18/3 27/28	SIPS	3 T; TR = 8500 ms, TE = 98 ms, 64 slices, voxel size = 2 × 2 × 2 mm^3^	Y	Cross-sectional	↓FA bilaterally in the corticospinal tract, ILF, IFOF	UHR: neurocognitive deficits restricted to executive function and motor dexterity, less severe than in schizophrenia Lower FA in the left ILF and IFOF correlated with poorer cognitive performance
9	von Hohenberg et al., 2014	28 UHR 1 UHR-P 27 UHR-NP 34 HC	20.6 ± 3.9 20.4 ± 4.0	14/10 16/18	SOPS, BSABS	3 T; TR = 9400 ms, TE = 84 ms, 75 contiguous axial slices matrix size = 128 × 128, voxel resolution 2 × 2 × 2 mm^3^	Y	Longitudinal	Changes in RD and MD lateralized to the right: ↑ MD in the right hemisphere (mainly SLF, superior and posterior corona radiata, PTR, posterior IC, splenium and body of CC, fornix, but also anterior limb of IC and cerebral peduncle) ↑ RD in PTR ↓ FA not statistically significant No significant changes in AD	
10	Bernard et al., 2015	26 UHR 21 HC	18.73 ± 1.78 17.71 ± 2.65	17/9 8/13	SIPS	3 T; TR = 9600 ms; TE = 86 mm; 72 slices; voxel size 2 × 2 × 2 mm^3^	Y	Longitudinal	Higher baseline FA in UHR in the hippocampal-thalamic tract FA changes not statistically significant (↓ FA in left hemisphere after 12 months)	Higher baseline FA correlated with positive symptom severity after 12 months
11	Schmidt et al., 2015	28 UHR 24 HC	25.42 ± 6.74 27.75 ± 4.59	18/6 10/14	BSIP, BPRS	3 T; TR = 9200 ms, TE = 95 ms, 54 axial slices, voxel resolution 2.5 × 2.5 × 2.5 mm^3^	Y	Cross-sectional	↑ FA in various regions of the brain; mostly right ATR, IFOF, SLF, UF, forceps major ↓ MD in various regions of the brain, esp. left SLF, ILF, IFOF, right ILF	FA positive correlation with (+) symptom severity in right SLF
12	Katagiri et al., 2015	41 UHR 7 UHR-P 34 UHR-NP 11 UHR-NN 23 UHR-NA 16 HC	20.71 ± 5.53 24.18 ± 7.88 23.35 ± 6.49 23.19 ± 2.86	1/6 9/25 8/8	SIPS/ SOPS	3 T; TR = 7668 ms, TE = 100 ms, matrix size 128 × 128; voxel resolution 1.02 × 1.02 × 5 mm^3^	Y	Longitudinal	↓ FA in genu and body of CC	UHR-NP: Change in FA correlates with changes in (+) symptom severity
13	Bakker et al., 2016	23 UHR 33 HC	24.3 ± 3.1 26.2 ± 5.5	15/8 22/11	SIPS	3 T; TR = 4834, TE = 94 ms, matrix size 112 × 112; 38 continuous slices; voxel size 2.05 × 2.05 × 3 mm^3^	Y	Cross-sectional	↑ AD bilaterally in ATR, left IFOF, left SLF, splenium and body of CC, and superior corona radiata ↑ RD and MD Areas of ↓ MD in CG No significant changes in FA	No links between FA, AD and RD and (+) or (-) symptom severity
14	Rigucci et al., 2016	27 UHR 10 UHR-P 17 UHR-NP 26 HC	23.2 ± 3.2 21.3 ± 2.6 24 ± 2.3	6/4 11/6 18/8	SIPS	1.5 T, TR = 9400 ms; TE = 9 ms; matrix size = 128 × 128; section thickness = 1.9 mm^3^	Y	Longitudinal	UHR-P: ↓ FA in left SLF and ILF, splenium and body of CC, forceps major, left corticospinal tract, left ATR, left IFOF ↑ RD in splenium and body of CC, forceps major, bilaterally ATR, corona radiata, right cortical-spinal tract Changes in MD and AD not statistically significant	
15	Wang et al., 2016	87 UHR 10 UHR-P 69 UHR-NP 37 HC	21.5 ± 3.6 22.3 ± 4.0	58/29 20/17	CAARMS	3 T, TR = 9600, TE = 107 ms, voxel size = 2.0 × 2.0 × 2.0 mm^3^	Y	Longitudinal	↓ FA in left cingulum, left CC, left UF, forceps minor, left IFOF, left SLF, left ATR ↓ AD in the CG and CC bilaterally No significant differences in MD or RD	↓ FA in left IFOF, left UF and left ATR correlated with greater symptom severity
16	Krakauer et al., 2017	45 UHR 45 HC	23.71 ± 4.65 23.80 ± 5.15	22/23 22/23	CAARMS	3T, TR = 7035 ms; TE = 68 ms, matrix size = 128 × 128 × 75; voxel dimensions = 1.88 × 1.88 × 2 mm^3^	Y	Cross-sectional	↓ FA in ILF, IFOF, ATR No significant changes in AD, MO, or RD Multivariate analysis of PLSC: - ↓ FA, AD and MO, ↑ RD in widespread regions of the brain	SOFAS correlate + with FA in left ILF and AD in left IFOF and right SLF, and with MO in right IFOF, left SLF, right ATR Multivariate analysis of PLSC: - more (+) and (-) symptoms and low levels of functioning associated with ↓ FA, AD, MO and ↑ RD; inverse correlation in several areas
17	Bernard et at., 2017	26 UHR 24 HC	18.65 ± 1.74 17.83 ± 2.50	18/8 11/13	SIPS	3 T; TR = 9600 ms; TE = 86 mm; 72 slices; voxel size 2 × 2 × 2 mm^3^	Y	Longitudinal	↑ FA in UHR at baseline	Positive correlation between FA and symptom severity (+) in thalamo-motor tract No other correlations
18	Saito et al., 2017	46 UHR 7 UHR-P 39 UHR-NP 16 HC	22.93 ± 6.46 23.19 ± 2.86	13/33 8/8	SIPS/ SOPS	1.5 T; TR = 7668 ms, TE = 100 ms, number of slices = 30; matrix size, 128 × 128; voxel resolution 1.02 × 1.02 × 5 mm^3^	N	Longitudinal	↓ FA in CC (greater in UHR-NP)	↓ FA in genu of CC correlates with (-) symptom severity
19	Krakauer et al., 2018	30 UHR 23 HC	24.07 ± 5.12 24.48 ± 5.81	13/17 13/10	CAARMS	3 T; TR = 7035 ms, TE = 68 msAcquired matrix size = 128 × 99 × 75; voxel dimensions = 1.88 × 2.41 × 2 mm^3^	Y	Longitudinal	↓ FA in left corticospinal tract, right thalamic radiation, left SLF After 12 months: No significant inter-group differences in FA changes, but ↑FA in SLF in UHR, in UF in HC	Positive correlation between FA change and age in SLF (but not in HC) No significant correlations between baseline FA and (+) or (-) symptom severity or level of functioning (SOFAS) after 12 months No significant correlation between change in FA and (+) symptom severity or level of functioning (SOFAS) Positive correlation between change in FA and (-) symptoms in left IFOF, anterior thalamic radiation and SLF
20	Whitford et al., 2018	40 UHR 59 HC	20.3 ± 4.0 21.4 ± 5.9	25/15 33/26	SIPS	3 T; TR = 9000 ms, TE = 84 ms, slices = 72; matrix = 128 mm × 128 mm, voxel size = 2 × 2 × 2 mm^3^	Y	Cross-sectional	No significant differences in FA or RD in arcuate fasciculus or pyramidal tract	
21	Kristensen et al., 2019	116 UHR 49 HC	23.8 ± 4.2 24.4 ± 3.4	55/61 22/27	CAARMS	3 T; TR = 7058 ms; TE = 68 ms, acquisition matrix = 128 × 99 × 75; voxel dimensions = 1.88 × 2.41 × 2 mm^3^	Y	Cross-sectional	Focal↓ FA, esp. in right SLF and CG ROI analysis: ↓ FA in right ATR, right fornix, stria terminalis, right SLF, and left tapetum	PLSC: Trend-level interaction between FA and cognitive performance Higher FA correlated with better cognitive performance (but not in HC)
22	Tang et al., 2019	50 UHR 50 HC	19.7 ± 4.6 19.2 ± 3.9	30/20 30/20	SIPS	3 T; TR = 15,800 ms, TE = 109 ms, 70 contiguous axial slices, voxel size = 2 × 2 × 2 mm^3^	Y	Cross-sectional	Globally ↓ FA ↓ FA in cellular tissue ↓ ADt No significant changes in FW No significant FA in cellular tissue or FW changes between UHR-P and UHR-NP TBSS: No significant changes in FW ↓FA in cellular tissue in CC, right anterior, superior, and posterior corona radiata, right and left SLF No significant differences in AD and RD in cellular tissue No significant differences between UHR-P and UHR-NP	Negative correlation between social functioning and FA as well as FA, AD, or RD in cellular tissue No correlation between FW and poorer social functioning No correlation between other clinical symptoms and FA, FW, or FA, AD, or RD in cellular tissue Positive correlation of FA in cellular tissue with age in HC, but not in UHR TBSS: No correlation between FA in cellular tissue and clinical symptoms, except for a significant correlation between FA in cellular tissue and deterioration in social functioning
23	Fitzsimmons et al., 2020	20 UHR 23 HC	21.08 ± 4.25 21.3 ± 3.67	13/7 12/11	SIPS	3 T; TR = 9400 ms, TE = 84 ms, 75 contiguous axial slices, matrix size = 128 × 128, voxel size = 2 × 2 × 2 mm^3^	Y	Cross-sectional	↓ FA in CG ↑ RD in CG ↑ trace of diffusion tensor in CG No significant difference in AD	No significant links between DTI and symptoms
24	Straub et al., 2020	18 UHR 19 HC	18.33 ± 0.59 18.39 ± 0.60	6/12 5/14	Wisconsin schizotypy scales, SIPS/ SOPS	3 T; TR = 5,400 ms, TE = 95 ms, 38 contiguous axial slices, voxel size of 1.6 × 1.6 × 3.0 mm^3^	Y	Cross-sectional	↓ FA in limbic network; ↓ in anterior right EC and right orbitofrontal-cortex-adjacent tracts No significant ↓FA in fronto-parietal cortex, anterior limb of IC, anterior corona radiata, EC, or forceps minor	Negative correlation between ↓FA in limbic network and (+) symptom severity
25	León-Ortiz et al., 2020	33 UHR 38 HC	19.55 ± 4.14 20.92 ± 3.37	26/7 28/10	SIPS	3 T; TR = 12,000 ms, TE = 70 ms, 60 slices, matrix 128 × 128; slice thickness = 2.6 mm	Y	Longitudinal	↓ FA in UHR-P in PTR, but not in UHR-NP	

Note: UHR = ultra-high risk for psychosis; UHR-P = ultra-high-risk subjects who developed psychosis; UHR-NP = ultra-high-risk subjects who did not develop psychosis (NN—without medication; NA—on antipsychotic medication); APSS = Adolescent Psychotic-like Symptom Screener; SIPS = Structured Interview for Prodromal Symptoms; SOPS = Scale of Prodromal Symptoms; CAARMS = Comprehensive Assessment of At-Risk Mental States; BSABS = Bonn Scale for the Assessment of Basic Symptoms; BSIP = Basel Screening Instrument for Psychoses; BPRS = Brief Psychiatric Rating Scale; PLSC = Partial least squares correlation; FA = fractional anisotropy; MD = mean diffusivity; AD = axial diffusivity; RD = radial diffusivity; SLF = superior longitudinal fasciculus; ILF = inferior longitudinal fasciculus; IFOF = inferior fronto-occipital fasciculus; UF = uncinate fasciculus; CB = cingulum bundle; EC = external capsule; IC = internal capsule; CC = corpus callosum; ATR = anterior thalamic radiation; PTR = posterior thalamic radiation; SU = substance use; AP = antipsychotics; (+)—positive symptoms; (-)—negative symptoms; ↑—increase in DTI parameter indicating the direction of WM changes; ↓—decrease in DTI parameter.

**Table 2:** Comparison of DTI indices between schizophrenia patients (SZ) and UHR individuals.

DTI Parameter	SZ	UHR
FA	↓ in SLF, ILF, IFOF, CB, CC, UF, AF, IC, fornix, corona radiata	↓ in SLF, ILF, IFOF, CB, CC, UF, PTR, ATR, EC, IC, forceps minor ↑ SLF, IFOF, UF, ATR, forceps minor
AD	↑ UF, fornix, corona radiata	↓ UF, fornix, CG, CC, cerebellar peduncle, corticospinal tract ↑ ILF, IFOF, CC, EC, IC
RD	↑ CC, UF, corona radiata, CB, fornix	↑ CC, UF, corona radiata, SLF, ILF, IFOF, EC, IC, PTR, CG, medial lemniscus, cerebellar peduncle, cerebral peduncle
MD	↑ ILF, AF, CC, UF, fornix	↑ SLF, corona radiata, PTR, IC, CC, CG, IC, cerebral peduncle ↓ SLF, ILF, IFOF

Note: DTI = diffusion tensor imaging; SZ = schizophrenia patients; UHR = ultra-high risk for psychosis individuals; FA = fractional anisotropy; AD = axial diffusivity; RD = radial diffusivity; MD = mean diffusivity; SLF = superior longitudinal fasciculus; ILF = inferior longitudinal fasciculus; IFOF = inferior fronto-occipital fasciculus; UF = uncinate fasciculus; CB = cingulum bundle; EC = external capsule; IC = internal capsule; CC = corpus callosum; ATR = anterior thalamic radiation; PTR = posterior thalamic radiation; AF = arcuate fasciculus.

## Data Availability

Not applicable.

## Supplementary Materials

The following are available online at https://www.mdpi.com/article/10.3390/jcm10112515/s1, Table S1: Quality assessment.
